# The Nurse and the Sick-Room

**Published:** 1887-07-09

**Authors:** 


### A Noble Profession: Nursing the Sick.

### THE NURSE AND THE SICK-ROOM.

By Miss Mollett.

(*Continued from p. 230, vol. ii.*)

"The cleanliness of a sick-room or ward is such an important item in the treatment of disease according to modern views, and the theoretical knowledge of the harm done to the sick by dirt and decay is so widespread, that I am continually amazed at the ignorance displayed in its removal, and that not only among untrained nurses, but among others who should know better. Now I know a hospital where, until quite recently, it was customary for the night-nurses to wash the soiled draw-sheets in the bath-room adjoining the ward, and afterwards to hang the often only half-cleansed sheets to dry in the ward where the patients were sleeping. It would require even less than an average knowledge of the laws of health to know that such a proceeding must have been bad for sick people; but it was an old custom that had been followed for long, and so was not abandoned without a struggle. That, of course, is an extreme case. But in a German book on nursing I was reading the other day, we are recommended, by way of keeping the beds perfectly clean, to change the bottom sheet *once a fortnight.*

"Even in otherwise well-managed hospitals, however, it is often marvellous how nurses, who have been carefully taught, and told again and again that infection lurks in minute germs, that half the disease in the world is theoretically preventible by strict cleanliness, still fall again and again into careless habits with regard to utter cleanliness in their patients and their surroundings. With regard to the actual cleaning of the ward, it is very usual to hear a modern probationer grumble at her share of the work, saying that she does not consider dusting and polishing, nursing, and she does not see why she should be made to do it. Then, unless carefully supervised, she will scamp the necessary cleaning, to turn to the more scientific and interesting part of her work. As to how much cleaning a nurse should do, that is a question that has not yet been finally settled. It is generally allowed that she should not be called upon to do the meaner housework, such as scrubbing, grate-cleaning, and polishing; but the amount of ward-work actually done by the nurses varies at different hospitals. Now, whether that amount be large or small, nothing is less to the nurse's credit than for her to be continually grumbling at her work and performing it inefficiently, on the virtual plea that she is too good for it. As to whether it is a good thing for her to have to give so much of her time to it, she may have a private opinion, if she likes; but, given a ward that she knows it is of the utmost importance to the health of her patients shall be clean, and herself as the only person to clean it, however smartly she may do the actual nursing, she is guilty of a grave neglect of duty to those in her charge, as well as to her superiors, if she does not do it properly. In these days it is the very rarest thing for a hospital nurse to be called upon to scrub floors; but should she be called upon to do so, she should made up her mind that it shall not be her fault if her patients have to lie in a dirty room.

"Cleanliness and ventilation are really one and the same thing, in so far as they both have the same end in view, namely, the purifying of the atmosphere of a sick-room; the one being the removal of all that might taint the atmosphere, the other of the atmosphere that has been tainted. The ventilation of a hospital, in other words, the rapidity with which foul air is removed from the wards, and fresh substituted, depends in a great measure on the good management of the nurses, but also to a very large extent on the arrangement of the building and its site. It was not left to modern times to discover the injurious effects of over-crowding sick people with insufficient means of ventilation. A document is extant, dated 1250, in which the master of a hospital in Regensburg, an Augustin Friar, writes, 'That the hospital is far too small, consequently there is not alone insufficient room for the sick poor, but amongst those that are admitted disease spreads from one to the other, shortening the lives of many. This is due to the faulty construction of the building, and the foulness of the air, which is poisoned by the reeking bodies of so many sick folk, crowded so closely together.' I do not know whether the Friar's representation was successful, and his hospital enlarged and improved. The old Xenadochia of which Miss Manson spoke, such as that veritable town of Basel, in which the sick lay separated in small houses, is the very *beau-idéal* of the modern reformer, who is opposed from a sanitary point of view to bringing together too many sick under one roof. Modern hygiene has decided that the pavilion system of building hospitals, by which a limited number of wards are arranged in separate blocks, which are thus surrounded by fresh air, is the best.

"Some American authors even go so far as to affirm that a hospital should be built so slightly that it can be entirely pulled down every ten or twenty years, and built again with fresh materials on another spot.

"The wards should be the width of the block, so that there can be a free cross ventilation, care being taken to leave a sufficient space between the windows for a bed to stand, so that it may not be, as in a modern hospital I know of, where the architect's design necessitated there being so many windows in the ward, that every other bed is perforce placed directly under one, to the great distraction of the nurse when she wishes to air the ward.

"Of the amount of cubic space required per bed in a ward, of the injurious effects of bad air, of the all-importance of good ventilation, and of all the details connected with it, so much has been said and written, that I will only mention very briefly that the minimum of space allowed to each patient should be 1,200 cubic feet, and that all the air contained in that space must be changed every two hours, to keep the ward sweet and wholesome. The best arrangement is that by which the air is being continually renewed without draught. Wherever patent ventilators are provided, of course they should be used, but they are not by any means always a success, and are seldom sufficient by themselves. I myself have a distinct prejudice in favour of the old-fashioned method of ventilating by well-placed and properly constructed windows and doors, and good fires which draw the foul air up the chimneys. A ward in which the nurse relies too exclusively on her ventilating apparatus is apt to be more than a little close. It is very seldom necessary to insist much to modern nurses upon the need of making their wards as bright and pretty as possible. A modern nurse is much more likely to err on the side of over-ornamentation. I am very fond of flowers, books, pictures, and, to a limited extent, of vases and birds in a ward, but always in moderation, carefully selected and well arranged. A ward should be bright, a ward should have well-chosen plants, not fading and drooping flowers, and good pictures; but it should not be overcrowded with gimcracks of all kinds, second-rate oleographs, cheap vases, and noisy canaries, or even, as a crowning abomination, a parrot that yells 'Pretty Poll' at inconvenient moments. Where there are too may ornaments in a ward, the dusting takes twice as long as there is any necessity for it to do, or else it is not properly done, and the ornaments only hide dirt and dirty corners. Noisy birds are simply unbearable in a medical ward, and can only just be tolerated in an accident ward. Personally, I should banish them from any ward in my charge; for, speaking from my own experience, when I was a probationer, I was ill, and they warded me. I distinctly remember the kind of agony I endured from two pet canaries of sisters, that hung opposite my bed and sang shrill duets throughout the day. Birds that do not sing are, I think, the best for a ward, for the notes of most birds become, after a time, monotonous and irritating to sick people.

"I have now said something about discipline, quiet, ventilation, cleanliness, and so forth; but I have not yet touched on what lies at the root of our present nursing system, what is, in fact, the seed from which all this modern enthusiasm for nursing has sprung. As Miss Manson explained at length in her paper, nursing in old times was essentially a Christian profession based on Christian doctrine, the Christian honoured in the leper the image of the Divine, his brother in the Lord; the sick were tended for the love of Christ; nurses were men and women filled with religious enthusiasm. The motto, 'God wills it,' which drove the knights of the middle ages to the Crusades, made them indeed for a short time, nurses and protectors of the poor and abandoned. Only under some religious influence was the care of the sick undertaken. Religious stimulus was absolutely necessary to its vitality; it was not common humanity which in those days drove men and women to nursing, but humanity practised in obedience to a creed which raised those who practised self-denial and good deeds to the ranks of the saints, and which reckoned the care of the sick among the primary good deeds. The era of religious enthusiasm was, if I may call it so, also the era of the first golden age of nursing. Nursing has still for the true Christian a sanctity upon which it is not necessary to touch; but what must it have had for those whose natures, less complex than ours, were entirely filled with one great all-pervading enthusiasm, which would thus find a legitimate channel?

"But when that enthusiasm died, or only flickered feebly in individuals, the stimulus it gave failed, and nursing gradually became purely mechanical drudgery, whose connection with the science of medicine was not acknowledged, because not understood, and whose votaries worked almost wholly and solely for a living: rarely for any higher motive. In such hands sick-nursing became a calling, the members of which not only ranked very low in the social scale, but were often viewed, and justly so, with much of that disgust which is shown in Dickens's famous sketch. They had not only the usual faults of women, such as are present in many of the 'undesirable nurses' of the present day; but they had others, which are now happily rare, and are becoming more so every day, but which were then supposed to be inseparable from hospital nurses. I read the other day a pamphlet, written in 1865, in which the establishment of nursing on the 'new principle' in a large hospital was described, where, after investigation, as a preliminary step, every nurse in the building but four had to be expelled for disorderly conduct and drunkenness!

"To bring a new spirit into a profession that had sunk so low, required a strong fresh stimulus, but the religious enthusiasm of the nineteenth century was not found adequate to supply it. So, when Florence Nightingale founded the great system of modern nursing, she appealed to our common humanity and our sense of justice, and she founded her system by practical common sense on the laws of science. We do not nurse the poor in the narrow spirit of a penance, much as we might walk with peas in our shoes to some shrine; we do it because it is a pleasure to us to nurse, because we love nursing. Nurses no longer ask to be enshrined with saints; they do not call on anyone to admire their devotion and their sacrifices; they honestly own to the world that it is no sacrifice to them to nurse, for they love their work. The scientific training they have received has taught them to appreciate its interests. A good nurse finds no dreariness nor sameness in the hospital wards that unfold before her a life of such varied interest and unwearying well-doing. Nursing, like all the other noble efforts towards well-doing of this last half of the nineteenth century, has its root in the great modern doctrine of humanity, of that common humanity which knits together all who are moved by compassion towards the sick and the suffering, whatever their religious denomination, and whose efforts are directed as much to raise as to relieve the unfortunate."

### HOSPITAL BOX AND COX.

THE Middlesex Hospital was the first hospital which had separate cubicles for the day and night nurses. Formerly, at this institution and at others, the day-nurses used to sleep in the night-nurses' beds, and the night-nurses in the day-nurses' beds, an arrangement perhaps economical in character, but one certainly not conducive to the comfort or the health of the nurses. Does any reader know of a hospital where the old system, as above described, yet prevails?

